# Pediatric Tuberculosis in North Kivu: The Urgency of a Targeted Response to a Forgotten Childhood Disease

**DOI:** 10.1002/puh2.70311

**Published:** 2026-07-03

**Authors:** Germain Cebweru Murhola, Christian Tague, Dujardin Makeda, Joshua Ekouo, Hermann Yokolo, Aymar Akilimali, Criss Koba Mjumbe

**Affiliations:** ^1^ Faculty of Medicine Université Libre des Pays des Grands Lacs Goma DR Congo; ^2^ Department of Research Medical Research Circle (MedReC) Bukavu DR Congo; ^3^ Centre de Recherche et d'Appui aux Systèmes de Santé (CRASS) Goma DR Congo; ^4^ Faculty of Medicine University of Lubumbashi Lubumbashi DR Congo

**Keywords:** children, North Kivu, tuberculosis

## Abstract

Pediatric tuberculosis remains a neglected health emergency, particularly in fragile contexts such as North Kivu province in the Democratic Republic of Congo. While more than one million children contract tuberculosis each year worldwide, the majority of cases remain underdiagnosed, resulting in preventable morbidity and mortality. In North Kivu, armed conflict, massive displacement, chronic malnutrition, overcrowding, and a weak health system create fertile ground for the spread of tuberculosis and complicate child care. Limited access to diagnostic tools (radiography, GeneXpert), insufficient Bacillus Calmette–Guérin (BCG) vaccination coverage, and a lack of appropriate pediatric drug formulations exacerbate the situation. National and international tuberculosis control strategies remain largely focused on adults, leaving children on the margins of policies and interventions. This editorial calls for an urgent and targeted response, including active contact tracing, improved access to diagnostic tools, the availability of appropriate medications, community outreach, and the involvement of humanitarian actors. Coordinated and context‐specific action is essential to ensure that children in North Kivu no longer remain the invisible victims of a preventable and curable disease.

## 1 Introduction

Tuberculosis remains one of the leading causes of preventable childhood mortality worldwide, with more than one million pediatric cases occurring each year. In 2023, tuberculosis is estimated to have affected approximately 10.8 million people worldwide, including 6 million men, 3.6 million women, and 1.3 million children. Present in all countries and at all ages, this infectious disease remains both preventable and curable [1, 2]. In the Democratic Republic of Congo (DRC), a country ranked among the 30 most endemic, the burden of tuberculosis remains high, particularly in the east of the country where health conditions are particularly poor [3]. In the North Kivu province, plagued by chronic armed conflict, massive population displacement, and a partial collapse of the health system, the situation is alarming. Yet, within this protracted humanitarian crisis, one reality remains hidden: tuberculosis in children. Often underdiagnosed, neglected in public policies, and absent from intervention priorities, it evolves in the shadows. However, despite the high burden of tuberculosis in this context, pediatric tuberculosis remains insufficiently documented and largely overlooked in both scientific literature and public health responses. Existing studies in the DRC and similar humanitarian settings have predominantly focused on adult tuberculosis or general epidemiological trends, with limited attention to the specific challenges of diagnosis, reporting, and management in children. This creates an important knowledge gap regarding the real burden and the specific vulnerabilities of children affected by tuberculosis in conflict‐affected areas such as North Kivu. This editorial aims to draw the attention of decision‐makers, health professionals, and humanitarian partners to this silent emergency, in order to foster a response adapted to the specific needs of children.

## 2 Methodology

This analysis is based on a narrative review of the scientific literature on pediatric tuberculosis, with a particular focus on the context of North Kivu province in the DRC. The main databases consulted were PubMed, WHO IRIS, UNICEF, and the Global TB Report, supplemented by reports from non‐governmental organizations (MSF, UNHCR, ICRC) and documents from the National Tuberculosis Control Program (PNLT‐DRC). The following MeSH terms and keywords were used in various combinations: “tuberculosis,” “pediatric tuberculosis,” “childhood tuberculosis,” “North Kivu,” “Democratic Republic of Congo,” “sub‐Saharan Africa,” “conflict settings,” “humanitarian crisis,” “diagnosis,” “treatment,” and “prevention.” Publications from 2000 to 2025 were considered, with priority given to studies published within the last 10 years. Inclusion criteria comprised original research articles, systematic reviews, meta‐analyses, WHO and UNICEF technical reports, and institutional documents directly relevant to pediatric tuberculosis in fragile or conflict‐affected contexts. Articles in English and French were included. Grey literature from international organizations (WHO, UNICEF, MSF, UNHCR) was also consulted to supplement peer‐reviewed data, given the scarcity of locally produced evidence specific to North Kivu. Approximately 50 documents were screened, of which 19 were retained as references based on their relevance, methodological quality, and direct applicability to the subject.

## 3 A Forgotten Disease: The Silent Burden of Tuberculosis in Children

Pediatric tuberculosis is a silent global emergency often ignored by health policies. According to WHO data, approximately 1.1 million children under 15 years of age develop tuberculosis each year, of which more than 225,000 die, especially among the youngest children [4]. Less than half of cases are actually detected and reported, revealing a significant diagnostic deficit, particularly in children under 5 years of age. This deficit and diagnostic obstacles are due to multiple particularities that children may present, including nonspecific symptoms (intermittent cough, fever, and growth retardation) and difficulties in distinguishing them from other childhood pathologies. Moreover, the paucibacillary nature of the infection makes microscopic tests insensitive, and sputum and gastric aspirate samples remain difficult to perform in children [4, 5, 6].

Limited resources are also one of the major obstacles to diagnosing the disease in developing countries, such as the DR Congo, particularly North Kivu, where access to certain advanced diagnostic tests, such as chest x‐rays or molecular tests like GeneXpert, remains very limited in some cities. In addition, health professionals are often insufficiently trained to specifically detect and manage tuberculosis in children [5, 6]. These shortcomings lead to major diagnostic delays, which favor the onset of severe forms of the disease at a stage where early management could have significantly improved the prognosis. They thus directly contribute to avoidable pediatric mortality, which is still too high in these regions [7]. Faced with this critical situation, the World Health Organization (WHO) recommends the use of proactive screening algorithms, even at the cost of a certain overdiagnosis, considering that it is better to treat preventively than to let children die due to the lack of formal microbiological confirmation [5].

## 4 North Kivu: A Breeding Ground for Propagation

Pediatric tuberculosis in North Kivu, DRC, represents a neglected yet critical health challenge exacerbated by several factors. The region's protracted conflict has created conditions conducive to TB transmission among children, including malnutrition, overcrowding, HIV prevalence, population displacement, and weak healthcare infrastructure [5, 6]. Malnutrition is highly prevalent; nearly half of the child population suffer from chronic malnutrition, significantly elevating the risk of TB and perpetuating a vicious cycle where TB worsens nutritional status. Overcrowded living conditions and frequent displacement in conflict zones further amplify exposure, often to adults with active TB exposure, including multidrug‐resistant TB (multidrug‐resistant tuberculosis [MDR‐TB]). MDR‐TB remains a concern, with the limited availability of molecular diagnostic tools such as GeneXpert MTB/RIF, essential to detect resistant strains, distributed sparsely across health zones [8, 9].

The Bacillus Calmette–Guérin (BCG) vaccine coverage necessary for preventing childhood TB remains inadequate. Vaccine shortages reported in regions of North Kivu seriously hinder immunization efforts, leaving many vulnerable children unprotected. The scarcity of pediatric‐specialized healthcare services compounds the difficulty in diagnosing pediatric TB, as clinical diagnosis is often necessary due to paucibacillary disease and limitations of available tests. Introduction of adapted pediatric TB diagnostic algorithms incorporating molecular tests on gastric aspirates, such as Xpert MTB/RIF, has improved diagnosis and treatment initiation rates, though broader implementation is needed [9, 10].

## 5 A Response That Forgets the Youngest

Tuberculosis control in North Kivu remains largely focused on adults, leaving children in the shadows of national and international programs. Despite WHO recommendations, pediatric care remains hampered by insufficient child‐friendly drug formulations, limiting adherence and therapeutic efficacy [11]. Active contact screening activities, essential for early identification of infected children, remain sporadic and poorly integrated into field strategies [12]. This situation is compounded by the lack of clear and harmonized protocols for monitoring exposed children, leading to diagnostic delays and avoidable morbidity [13]. Addressing these gaps requires policy adaptation, dedicated funding, and systematic integration of pediatric screening into all levels of care, so that children cease to be the invisible victims of a curable disease. These gaps are not unique to North Kivu: Globally, children account for only 6% of notified TB cases despite representing an estimated 11% of the disease burden, reflecting a systemic underdiagnosis driven by limited access to appropriate diagnostic tools and inadequately trained health workers [14, 15]. Evidence from sub‐Saharan African settings indicates that systematic contact investigation of child household contacts of smear‐positive index cases can increase pediatric case detection by up to 30% [16]. The adoption of WHO‐recommended pediatric fixed‐dose combination tablets, designed to improve palatability and dosing accuracy in young children, remains insufficient in DRC despite their proven impact on treatment adherence and outcomes [17]. Furthermore, MDR‐TB represents a growing threat in conflict zones: Children exposed to adults with MDR‐TB are at high risk of acquiring resistant strains, yet appropriate second‐line pediatric regimens are virtually unavailable in North Kivu [18, 19]. These findings collectively underscore that the marginalization of children in TB control programs is both a humanitarian failure and a missed epidemiological opportunity.

## 6 Call to Action: For an Integrated and Urgent Pediatric Strategy

Since tuberculosis treatment is long‐term, we observe many cases of treatment abandonment, this being linked to multiple factors such as the displacement of populations fleeing the war, the lack of information among the population and shortages of medication stocks. Added to this problem is the lack of compliance with the vaccination schedule.

Overcrowding is a key factor in the transmission and spread of tuberculosis, yet it is also fueled by the living conditions in IDP camps. NGOs, the government, and other partners should take this into account when building homes for populations affected by war.

Educating parents of sick children about the danger of TB and the importance of not abandoning medication because the latter maintains resistance to anti‐tuberculosis drugs, integrating complementary tests such as Ziehl–Nielson staining or GeneXpert into routine check‐ups, especially in patients living in areas with a high prevalence of tuberculosis and high promiscuity.

Community participation in the fight against tuberculosis by involving community leaders in raising awareness about ways to prevent tuberculosis and identifying people under treatment to prevent cases of treatment abandonment could contribute to reducing TB mortality.

Provide pediatric medications and simplify protocols.

## 7 Conclusion

Pediatric tuberculosis in North Kivu represents a public health emergency that remains too silent. In a context marked by armed conflict, malnutrition, overcrowding, and a weakened health system, children remain the invisible victims of a disease that is nevertheless preventable and curable. Faced with this burden, it is imperative to develop a response specifically adapted to pediatric needs, integrating active screening, accessible diagnostic tools, medications formulated for children, and sustainable community strategies. The fight against tuberculosis in children cannot succeed without collective responsibility: The state, NGOs, donors, and health professionals must act together to ensure that tuberculosis ceases to be a forgotten disease of children in North Kivu.

## 8 Limitations of the Study

This article has several limitations that should be noted. First, the lack of recent, disaggregated local data on pediatric tuberculosis in North Kivu complicates accurate analysis of the true burden among children. The unstable security context, marked by armed conflict, also hinders the collection of reliable and up‐to‐date data. Furthermore, the scarcity of locally conducted pediatric clinical studies limits the generalizability of findings from other regions. Because the approach adopted here is narrative and non‐systematic, it may introduce source selection bias.

## Author Contributions

**Dujardin Makeda**: writing – original draft, writing – review and editing. **Germain Cebweru Murhola**: conceptualization, writing – original draft, writing – review and editing. **Hermann Yokolo**: writing – review and editing, writing – original draft. **Criss Koba Mjumbe**: supervision, project administration, writing – review and editing, validation. **Aymar Akilimali**: writing – review and editing, writing – original draft, investigation. **Joshua Ekouo**: writing – review and editing, writing – original draft. **Christian Tague**: conceptualization, investigation, writing – original draft, writing – review and editing, methodology, project administration, supervision.

## Funding

The authors have nothing to report.

## Ethics Statement

The authors have nothing to report.

## Consent

The authors have nothing to report.

## Conflicts of Interest

The authors declare no conflicts of interest.

## Data Availability

No datasets were generated or analyzed during the current study.
